# Compromised barrier integrity of human feto-placental vessels from gestational diabetic pregnancies is related to downregulation of occludin expression

**DOI:** 10.1007/s00125-020-05290-6

**Published:** 2020-10-01

**Authors:** Stephany Daniela Villota, Maria Toledo-Rodriguez, Lopa Leach

**Affiliations:** School of Life Sciences, University of Nottingham, Nottingham, UK

**Keywords:** Gestational diabetes, miR-181a-5p, *OCLN* splice variants, Vascular leaks

## Abstract

**Aims/hypothesis:**

Reduced occupancy of junctional occludin is a feature of human placental vessels in the diabetic milieu. However, the functional consequence of this and whether this loss is due to differential expression of occludin splice variants is not known. Our study aimed to investigate the effects of gestational diabetes mellitus (GDM), and its treatment, on endothelial junctional integrity, gene and protein expression of occludin splice variants, and potential regulation of expression by microRNAs (miRNAs).

**Methods:**

Term placentas were obtained from normal pregnancies (*n* = 21), and pregnancies complicated by GDM where glucose levels were controlled by diet (*n* = 11) or metformin (*n* = 6). Gene and microRNA (miRNA) expression were determined by quantitative real-time PCR; protein expression by immunoblotting; endothelial junctional occupancy by fluorescence microscopy and systematic sampling; and paracellular leakage by perfusion of placental microvascular beds with 76 *M*_r_ dextran. Transfection studies of miRNAs that target *OCLN* were performed in HUVECs, and the trans-endothelial electrical resistance and tracer permeability of the HUVECs were measured.

**Results:**

All three predicted *OCLN* gene splice variants and two occludin protein isoforms were found in human placental samples. In placental samples from diet-controlled GDM (d-GDM) pregnancies we found a lower percentage of conduit vessels showing occludin immunoreactivity (12%, *p* < 0.01), decreased levels of the fully functional occludin isoform-A protein (29%), and differential gene expression of *OCLN* variant 2 (33% decrease), variant 3 (3.3-fold increase). These changes were not seen in samples from the group with metformin-controlled GDM. In d-GDM placentas, increased numbers of conduit microvessels demonstrated extravasation of 76 *M*_r_ dextran (2.0-fold). In d-GDM expression of one of the five potential miRNAs targeting *OCLN*, miR-181a-5p, expression was 2.1-fold that in normal pregnancies. Experimental overexpression of miR-181a-5p in HUVECs from normal pregnancies resulted in a highly significant downregulation of *OCLN* variant 1 (69%) and variant 2 (46%) gene expression, with decreased trans-endothelial resistance (78%) and increase in tracer permeability (1.3-fold).

**Conclusions/interpretation:**

Downregulation of expression of *OCLN* variant 2 and the fully functional occludin isoform-A protein are a feature of placentas in d-GDM pregnancies. These may be behind the loss of junctional occludin and the increased extravasation of exogenous dextran observed. miR-181a-5p was in part responsible for the downregulation of occludin in placentas from d-GDM pregnancies. Induced overexpression of *miR-181a-5p* compromised the integrity of the endothelial barrier. Our data suggest that, despite good glucose control, the adoption of lifestyle changes alone during a GDM pregnancy may not be enough to prevent an alteration in the expression of occludin and the subsequent functional consequences in placentas and impaired vascular barrier function in offspring.

**Figure:**
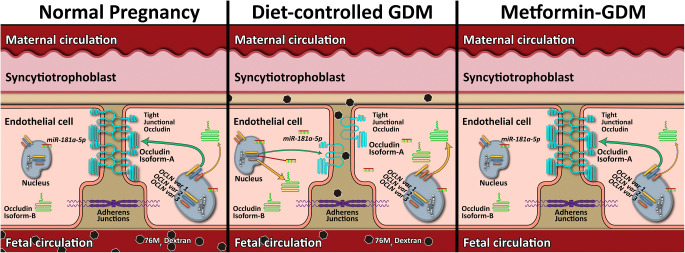
Graphical abstract

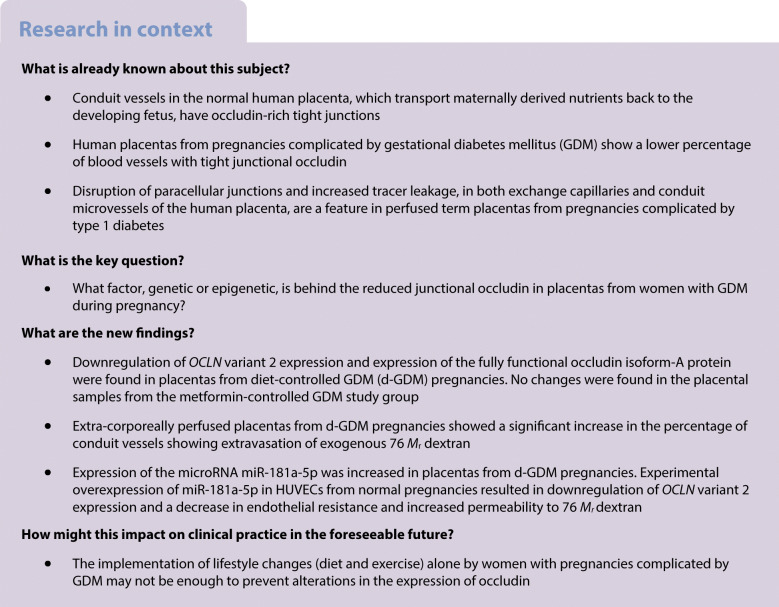



## Introduction

Gestational diabetes mellitus (GDM) is defined as a carbohydrate intolerance resulting in any degree of hyperglycaemia first recognised during the second or third trimester of pregnancy [1]. GDM increases the risk of several negative consequences for the mother and the infants, the most significant of which is a predisposition to develop metabolic syndrome and type 2 diabetes [2, 3]. Placental pathophysiology in GDM also includes increased insulin resistance, inflammation, increased glucose uptake, alterations of glucose transporters and endothelial dysfunction (including junctional integrity) [4, 5]. The latter may affect fetal programming of adult cardiovascular disease. Following the UK National Institute for Health and Care Excellence (NICE) guidelines, all women diagnosed with diabetes are advised to adjust their diet and adopt a physical exercise routine if they are planning a pregnancy or are pregnant [6]. Lifestyle changes are sufficient to control hyperglycaemia in 80–90% of women with GDM [7]. If a diet change is insufficient, metformin or insulin are used [6].

The placenta, a fetal end organ, forms a semi-permeable barrier between mother and fetus. It is composed of numerous chorionic villi containing fetal exchange capillaries, which pick up nutrients, and conduit microvessels, which transport solutes back to the fetus via the chorionic plate venous network and single umbilical vein [8]. The villous outer lining is a single multinucleated layer of syncytiotrophoblast, which is in direct contact with maternal blood. The haemodynamics here will influence solute uptake and oxygenation [8]. Hydrophilic solutes have to cross both the syncytiotrophoblast and the endothelium to reach the fetal blood [9]. The endothelial layer acts as a barrier in series by containing well-defined junctions (adherens and tight junctions). Disruption here leads to reduced junctional restrictiveness, altered cleft dimensions [10], increased solute transit time and paracellular vascular leaks [11, 12], all impacting on optimal nutrient delivery to the developing fetus and impaired placental barrier function. In the blood–brain barrier, tight junction disruption has been shown to lead to changes in the efflux/influx of hydrophilic solutes [13]. Disruption of junctions by histamine can increase arterial flow and permeability of venules in the mouse ear [14]. Similar mechanisms may be at play in the human placenta.

A perfusion study using freshly delivered term placenta from pregnancies complicated with type 1 diabetes reported an increase in vascular leakage in all vessels of the microvascular bed [15]. This was correlated with perturbations of junctional proteins, phosphorylation of VE-cadherin and increased vascular endothelial growth factor (VEGF) protein expression [15]. Likewise, placentas from pregnancies complicated by GDM have shown loss of junctional proteins [5, 16]. However, studies into the genetic or epigenetic regulation of these transmembrane junctional proteins were not performed.

**Fig. 1:**
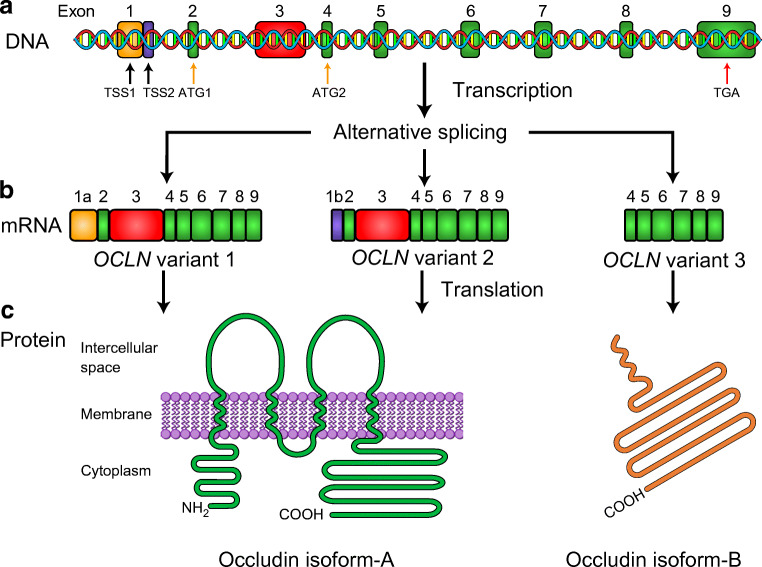
Schematic representation of human occludin mRNA and protein products. (**a**) Gene structure of human *occludin* includes nine exons. Alternative splicing generates three variants. Alternative transcriptional start sites (TSS1 and TSS2, black arrows) and translational start sites (ATG1 and ATG2, orange arrows) in exons 1, 2 and 4 are indicated. The stop codon within exon 9 is also indicated (TGA, red arrow). (**b**) Three mRNA variants are generated. ATG1 forms *OCLN* variants 1 and 2, each of these two variants has a different transcriptional start site. ATG2 generates *OCLN* variant 3. (**c**) *OCLN* variant 1 and 2 translate into occludin isoform-A; a fully functional, membrane protein. *OCLN variant 3* translates into occludin isoform-B, a truncated, cytoplasmic protein

Occludin is a tight junctional strand protein that interacts with scaffolding proteins in the zonula occludens to adjoin cell–cell overlap of paracellular clefts and maintain the integrity of tight junctions. Loss of occludin and disrupted tight junctions are a feature of diabetic retinopathy [17], and this feature has also been reported in placental vessels in pregnancies complicated with GDM [5]. Whether differential expression of splice variants of the gene encoding occludin (*OCLN*) is behind these changes requires investigation. The human *OCLN* gene includes nine exons (Fig. 1a) and alternative splicing produces different mRNAs [18]. The NCBI database reports three predicted splice variants (Fig. 1b): *OCLN* variant 1 (NM_002538.3), variant 2 (NM_001205254.1) and variant 3 (NM_001205255.1). Differences in exon conformation and promoter regions lead to the translation of two different protein isoforms (Fig. 1c): occludin isoform-A (NP_002529.1) and isoform-B (NP_001192184.1). *OCLN* variant 1 and variant 2 translate into occludin isoform-A (~60 kDa), a complete and functional protein that can localise to the membranes of tight junctions and participate in homophilic binding. *OCLN* variant 3 translates into a truncated cytoplasmic occludin, known as isoform-B (~30 kDa). The function of isoform-B has not been described; however, a study using Madin-Darby Canine Kidney (MDCK) cells expressing chimeric occludin determined that a variant that lacks extracellular loops but presents the COOH-terminal cytoplasmic domain results in intracellular vesicular accumulation of all the scaffold of tight junction proteins [19].

Occludin expression can be epigenetically regulated. For example, the microRNAs (miRNAs) miR-18a-5p [20, 21], miR-181a-5p [22], miR-21-3p [23], miR-122-5p [24, 25], and miR-340-5p (DIANA database; http://diana.imis.athena-innovation.gr/DianaTools/index.php) have been reported to be expressed in human placenta and to target *OCLN* expression in different tissues. The relationship between each miRNA, human placenta, *OCLN* expression and GDM has not previously been analysed.

The aim of this study was to determine the effects of GDM on the integrity of the feto-placental vessels and the expression of occludin. We hypothesised that the type of treatment used to control glucose levels in pregnancies complicated by GDM influences the level to which the placental vasculature is affected.

## Methods

### Study population

Pregnant women, with singleton pregnancies, were recruited at the Queen’s Medical Centre, Nottingham University Hospitals NHS Trust, UK. This study had the approval of the East Midland Nottingham 2 – Health Research Authority (Ref: OG010101). It was carried out in accordance with The Code of Ethics of the World Medical Association (Declaration of Helsinki).

Term placentas (*N* = 38; >37 weeks’ gestation) from elective Caesarean sections (C-sections) were collected after informed consent. We chose to collect placentas only from C-sections in order to minimise the effects of post-parturition hypoxia and ischaemia and obtain placentas that had not received labour signals. For protein localisation, protein expression and gene expression analysis, placental samples were obtained from three groups: normal pregnancies (*n* = 9), pregnancies complicated with GDM treated by a change of diet (*n* = 7) (d-GDM) or by metformin (*n* = 6) (m-GDM) (Table 1). For tracer leakage analysis, term placentas were collected from normal pregnancies (*n* = 4) and d-GDM pregnancies (*n* = 4) (Table 2). Transfection studies, trans-endothelial resistance and permeability experiments were done with HUVECs isolated from normal pregnancies (*n* = 8).

**Table 1:** Clinical characteristics of the study population that had samples used for gene and protein expression

Characteristic	Normal (*n* = 9)	d-GDM (*n* = 7)	m-GDM (*n* = 6)	*p* value
Maternal age, years	34.67 (6.04)	30.57 (6.78)	31.83 (4.71)	0.390
Maternal BMI, kg/m^2^	25.71 (4.27)	29.20 (7.54)	32.38 (6.89)	0.145
Gestational age at delivery, weeks	38.69 (1.18)	39.17 (0.26)	39.40 (0.37)	0.236
HbA_1c_, mmol/mol	–	37.44 (3.99)	36.60 (2.97)	0.848
HbA_1c_, %	–	5.57 (0.36)	5.60 (0.34)	0.924
Placental weight, g	674.4 (108.9)	674.4 (119.4)	644.2 (98.4)	0.848
Baby weight, kg	3.49 (0.37)	3.54 (0.69)	3.62 (0.71)	0.924
Individual birthweight centile [sex]	47.15th [M] 76.31th [F] 62.16th [M] 95.67th [M] 93.48th [M] 88.55th [F] 29.82th [M] 46.28th [M] 98.96th [M]	93.51th [F] 54.28th [F] 30.92th [F] 28.40th [M] 99.96th [M] 66.66th [F] 67.69th [M]	80.82th [M] 91.68th [F] 99.94th [M] 43.02th [F] 15.77th [F] 71.76th [M]	0.857

Data are presented as mean (SD); *p* value calculated by one-way ANOVA

[F], neonatal sex female; [M], neonatal sex male

**Table 2:** Clinical characteristics of the study population that had samples used for the placental permeability assay

Characteristic	Normal pregnancy (*n* = 4)	d-GDM (*n* = 4)	*p* value
Maternal age, years	30.75 (3.76)	32.00 (7.53)	0.777
Maternal BMI, kg/m^2^	27.00 (2.83)	29.15 (3.37)	0.366
Gestational age at delivery, weeks	38.25 (0.96)	37.50 (1.30)	0.387
HbA_1c_, mmol/mol		38.50 (3.51)	
HbA_1c_, %		5.68 (0.32)	
Placental weight, g	817.8 (125.1)	898.5 (199.7)	0.519
Baby weight, kg	4.04 (0.49)	4.15 (0.31)	0.706
Individual birthweight centile [sex]	94.47th [M] 99.87th [M] 99.73th [F] 84.03th [F]	99.96th [F] 99.10th [M] 99.93th [F] 93.48th [M]	0.407

Data are presented as mean (SD); *p* value calculated by one-way ANOVA

[F], neonatal sex female; [M], neonatal sex male

Women in all groups were of similar age, had no vascular complications, pre-eclampsia or hypertension, were non-smokers and were not taking any other medications.

### Tissue preparation and sampling

Placentas and umbilical cords were taken immediately after delivery. Chorionic villous biopsies were taken from four quadrants, midway between the umbilical cord insertion and the placental rim. For immunostaining, fresh tissue was fixed in 1% (wt/vol.) paraformaldehyde (PFA), frozen in nitrogen-cooled isopentane and stored at −80°C. For protein extraction, tissue was snap-frozen and stored at −80°C. For RNA isolation, tissue was placed in RNAlater solution (Sigma-Aldrich, USA) and stored at −20°C. For tracer leakage analysis, placentas were immediately processed as described below.

### Systematic random sampling of junctional occludin in vascular profiles

An indirect immunofluorescence method was used [26]. Briefly, frozen placental sections were permeabilised with acetone and 0.1% Triton X-100, blocked with 5% normal human serum, and incubated with primary antibodies for occludin (2.5 μg/ml; rabbit polyclonal; catalogue no. 71-1500, Thermo Fisher, USA) and the endothelial marker platelet endothelial cell adhesion molecule 1 (PECAM-1) (2.5 μg/ml; mouse monoclonal; catalogue no. BBA7, R&D Systems, UK). Sections were washed thoroughly and incubated with the appropriate secondary antibodies: FITC-conjugated anti-rabbit (10 μg/ml; catalogue no. F0382, Sigma-Aldrich) and tetramethylrhodamine isothiocyanate (TRITC)-conjugated anti-mouse (10 μg/ml; catalogue no. T5393, Sigma-Aldrich). All antibodies were diluted in 0.1% (wt/vol.) BSA in 0.1 mol/l PBS.

Immunoreactivity in the sections was visualised (blind) using a Nikon microscope (LaboPhot-2, Nikon Instruments, UK). A systematic random sampling of fields of view was adopted and micrographs acquired. The number of vascular profiles showing immunoreactivity to occludin and PECAM-1 was counted using the unbiased ‘forbidden line’ counting principle [27]. No more than 150 vascular profiles were counted for each placenta to maintain sampling efficiency.

### Protein isolation and western blotting

Placental tissue was homogenised in protein lysis buffer (50 mmol/l Tris pH 8.0, 150 mmol/l NaCl, 0.5% deoxycholate, 1% Triton X-100, 0.1% [wt/vol.] sodium dodecyl sulphate [SDS]) plus 2 mmol/l phenylmethylsulfonyl fluoride (PMSF), a protease (Roche, Germany) and phosphatase inhibitors cocktails (Sigma-Aldrich). Protein concentration was measured using the DC Protein Assay (Bio-Rad, USA).

Total protein (40 μg) was separated on a 4–20% SDS-PAGE gel (Bio-Rad). Nitrocellulose membranes were incubated with primary antibodies: polyclonal rabbit anti-occludin (0.5 μg/ml; catalogue no. 71-1500, Thermo Fisher), and monoclonal mouse anti-β-actin (7 μg/ml; catalogue no. A531, Sigma-Aldrich). Secondary antibodies: goat anti-rabbit (catalogue no. 926-32211, LI-COR, Germany), and goat anti-mouse (catalogue no. 925-68070, LI-COR) diluted to 0.1 μg/ml. Membranes were scanned with an Odyssey Infrared Imaging System 3.0 (LI-COR). Quantification of bands was performed with ImageJ Software Version 2.0 [28]. Fluorescence intensity of occludin bands was normalised to their respective loading control. All antibodies were diluted in blocking buffer (5% [wt/vol.] milk (in 1× Tris-Buffered Saline, 0.1% Tween 20 detergent).

A similar protocol was used for VEGF-A with minor modifications: 20 mg of total protein and primary antibody polyclonal rabbit anti-VEGF-A (3.6 μg/ml; catalogue no. ab183100, Abcam).

### RNA isolation and qPCR

Total RNA was extracted using TRI Reagent (Sigma-Aldrich) and a TissueLyser LT (Qiagen, Australia) homogeniser. RNA quantity and quality were assessed using the NanoDrop spectrophotometer ND-2000 (Thermo Fisher).

Five μg of total RNA were reverse transcribed using SuperScript III Reverse Transcriptase (Invitrogen, USA) and random primers (Promega, USA). Quantitative real-time PCR (qPCR) reactions were performed in triplicate with SYBR Green JumpStart Taq ReadyMix (Sigma-Aldrich), and detection was performed using the Rotor-Gene 6000 cycler (Corbett Research, Mortlake, NSW, Australia). The thermal profile included 95°C for 10 min, 40 cycles of 95°C for 15 s, annealing temperature (specific of each primer) for 30 s, and 72°C for 40 s (Table 3). Gene expression was normalised to a geometric average of three reference genes (*YWHAZ*, *TBP*, *SDHA*) [29]. The relative expression changes were determined using the relative quantification (${2}^{-{\Delta  \Delta  \mathrm{C}}_{\mathrm{t}}}$) method [30].

**Table 3:** Primer sequences

Gene	Sequence	Primer concentration (nmol/l)	cDNA dilution	Annealing temperature (°C)
*YWHAZ*	F: 5′-TGGCTCGAGAATACAGAGAGAA-3′ R: 5′-TGGGGATCAAGAACTTTTCCAA-3′	200	1:256	58
*TBP*	F: 5′-CCTAAAGACCATTGCACTTCGT-3′ R: 5′-TTCGTGGCTCTCTTATCCTCAT-3′	400	1:64	62
*SDHA*	F: 5′-GTGCCGTGGTGTCATCGC-3′ R: 5′-CTGGTGTGGGCAGACGTG-3′	300	1:256	60^a^
*OCLN* variant 1	F: 5′- TGAATTGGTCACCGAGGGAG-3′ R: 5′- TAAACCAATCTGCTGCGTCCTA-3′	600	1:8	60^a^
*OCLN* variant 2	F: 5′- GGCGAGCGGATTGGTTTAT-3′ R: 5′- AAGGAGGTGGACTTTCAAGAGG-3′	600	1:64	58^a^
*OCLN* variant 3	F: 5′-CCTTACAGGCCTGATGAATTGC-3′ R: 5′-TGTCCATCTTTCTTCGAGTTTTC-3′	F 300 / R 800	1:8	62^a^
*VEGF-A*	F: 5′- ATCTTCAAGCCATCCTGTGTGC-3′ R: 5′- TATGTGCTGGCCTTGGTGAG-3′	700	1:8	60^b^

^a^1 mmol/l betaine was needed for the specificity of the qPCR reaction

F, forward; R, reverse

### Placental perfusion

A well-established dual-perfusion procedure was employed [9, 11, 12, 27]. Briefly, immediately after delivery by C-section, the placenta was transferred to a 37°C chamber with the umbilical cord kept clamped to prevent loss of blood and collapse of feto-placental vessels. Within 20 min, a chorionic vein and an artery supplying a chosen cotyledon were cannulated to establish the fetal circulation. Fetal arterial inflow and venous outflow was measured to ensure they were the same. Once venous outflow showed clear blood-free perfusate (several minutes), the placenta was inverted and the cotyledon clamped in a Perspex chamber to isolate it from the rest of the placenta. The independent maternal circulation was simulated by inserting five nasogastric tubes (equally spaced, one per lobule) into the intervillous space through the basal plate of the cotyledon and drained through an exit tube in the chamber. Fetal and maternal circulations were connected to peristaltic pumps providing a constant flow of 20 ml/min to the maternal circulation and a 5 ml/min flow to the fetal circulation. Maternal and fetal perfusion pressures were monitored continually by placing pressure transducers just prior to arterial inflows of both circuits. A maternal blood pressure of 18–20 mmHg and a fetal blood pressure of 40–80 mmHg was accepted as normal. Increases above these ranges led to abandonment of the experiment (one in five failure rate). Flow rates and perfusion pressures were visualised continually with ML866/P PowerLab 4/30 recording unit and LabChart Pro software (ADInstruments, UK).

A 20 min open circuit equilibration period was performed to reverse any post-parturition hypoxic changes [9]. During this period, both circuits were irrigated with oxygenated Medium 199 (Sigma-Aldrich), with added sodium bicarbonate (2.2 g/l), albumin (5 g/l), high-molecular-weight dextran (2000 *M*_r_; 8 g/l) and heparin (5000 IU/l) (final pH 7.2–7.4). After equilibration, fetal oxygenation was stopped, 76 *M*_r_ dextran conjugated with TRITC (1 mg/ml; Sigma-Aldrich) was added as a bolus into the closed fetal circulation for 10 min. The maternal circuit remained open, oxygenated and tracer free. Finally, 1% (wt/vol.) PFA was introduced into the fetal circuit, the clamped Perspex chamber was removed to prevent bulk flow and the cotyledon perfusion fixed for 30 min. The cotyledon was excised, weighed (~50 g) and vertical slices (basal plate to chorionic plate) from fixed, blanched lobules only were taken for a further immersion fixation (1 h). Biopsies (villous trees) were frozen in nitrogen-cooled isopentane and stored. Cryosections were obtained, PECAM-1 detected by immunocytochemistry, and micrographs acquired by systematic random sampling of fields of view.

### Tracer leakage sampling

The vascular profiles displaying tracer leakage (hotspots) were counted (blinded) using unbiased ‘forbidden line’ counting principle [12, 15, 27]. To maintain efficiency, between 100 and 200 vessels were counted in each placenta from each group. The number of vessels with hotspots (in terminal, intermediate and stem villi) was subsequently expressed as a percentage of total vessels counted (all vessels in the field of view). The percentages of conduit vessels in stem and intermediate villi from normal and d-GDM groups were analysed for this study.

### Quantification of microRNA expression

Total RNA (5 ng) was reverse transcribed using a miRCURY LNATM Universal RT microRNA PCR Kit (Exiqon, Denmark) per manufacturer’s instructions. miRNAs that target *OCLN* expression were selected based on literature and a bioinformatics search using three databases: miRBase (www.mirbase.org), miRTarBase (http://mirtarbase.mbc.nctu.edu.tw) and DIANA (http://diana.imis.athena-innovation.gr/DianaTools/index.php). Five miRNAs were selected: miR-18a-5p, miR-21-3p, miR-122-5p, miR181a-5p and miR-340-5p. Exiqon primers were used for amplification (catalogue nos YP00204207, YP00204302, YP00205664, YP00206081 and YP00206068, respectively). qPCRs were conducted in triplicate using ExiLENT SYBR Green master mix (Exiqon). Detection was performed using the Rotor-Gene 6000 cycler; 95°C for 10 min, followed by 40 cycles of 95°C for 10 s, and 60°C for 1 min. Samples were diluted 1:80 before qPCR and 4 μl of the diluted samples were used in 10 μl reactions. U6 spliceosomal RNA gene was used as an internal control (catalogue no. YP00203907).

### Transfection of HUVECs with miRNA

HUVECs were isolated from normal pregnancies (*n* = 4) following a standardised protocol [31]. HUVECs were used for transfection of miR-181a-5p mimic between passages 2 or 3. Optimisation of transfection was done following manufacturer’s instructions (Dharmacon Research, USA). Briefly, HUVECs were seeded into 12 well plates at ~2.0 × 10^5^ cells/well in transcription medium (TM20, which is antibiotic-free M199 [catalogue no. 12340030, Thermo Fisher] with 20% FCS) and cultured overnight at 37°C in a humidified incubator with 5% CO_2_ in air. Each sample was treated under four conditions (*n* = 3 per condition, per sample): untreated control (TM20); transfection control (TM20 + transfection reagent); negative control (TM20 + transfection reagent + negative control mimic); miR-181a-5p transfection (TM20 + transfection reagent + miR-181a-5p mimic). Each reaction needed 0.75% DharmaFECT-1 transfection reagent (T-2001-01, Dharmacon), 15 nmol/l miRIDIAN miR-181a-5p mimic (C-300552-05-0005, Dharmacon), or 15 nmol/l miRIDIAN mimic negative control (CN-001000-01-05, Dharmacon). Cells were cultured for 24 h and the transfection medium was replaced with fresh TM20 for a further 24 h incubation. Finally, cells were washed and TRI Reagent was used to isolate RNA.

### HUVEC permeability assays

For assessment of trans-endothelial electrical resistance (TEER), HUVECs isolated from normal pregnancies (*n* = 4) were seeded in triplicate onto gelatine-coated 12 transwell inserts (catalogue no. CLS3493, Corning, USA). TEER was measured in triplicate before transfection and every 24 h for the next 3 days using an EVOM volt meter (World Precision Instruments, UK). TEER values were subtracted from blank TEER values (inserts without cells) and multiplied by the surface area of the transwell.

For tracer leakage, an aliquot of the same HUVECs used for TEER (*n* = 4) was seeded under similar conditions. After HUVECs were transfected (48 h), medium was replaced with phenol-free M199 (catalogue no. 11043023, Thermo Fisher), apical 0.5 ml, basal 1.5 ml, ensuring same level in inner and outer well. FITC-conjugated dextran (molecular weight 76,000; 1 mg/ml; Thermo Fisher) was added to the apical chamber. Fifty microlitres of medium from the basal chamber were collected every 30 min for 2 h; the volume taken was replaced to ensure hydrostatic pressure remained balanced after each sample collection. Tracer leakage was measured by a fluorescence microplate reader (Labtechnologies FLUOstar Galaxy Microplate Reader, UK) at an emission/excitation wavelength of 485/520 nm. The concentration of tracer in each sample was calculated via linear regression of a standard curve. Absolute permeability (cm/s) was calculated as the ratio of the tracer leakage to the concentration gradient as follows [32]:

$$ P=\frac{\left[C(t)\hbox{--} C\left({t}_0\right)\right]\times V}{A\times t\times {C}_0} $$

Where *C*(*t*) and *C*(*t*_0_) are the tracer concentrations (μg/ml) at 120 min and 0 min, respectively, estimated by linear regression; *V* is the volume in the lower compartment (1.5 cm^3^); *A* is the surface area of the transwell membrane (1.1 cm^2^); *t* is the duration of the flux (7200 s); and *C*_0_ is the initial concentration of the tracer on the apical side (1000 μg/ml).

### Statistical analysis

Data are expressed as mean ± SEM. Data were tested for normality of the distribution by the D’Agostino–Pearson test with a confidence interval of 95%. Study population, protein localisation, protein expression, placental perfusion, miRNA expression, TEER and HUVEC tracer leakage were normally distributed. Statistical comparisons between two groups were performed by independent samples *t* test, and among the three groups by one-way ANOVA. Results from gene expression were not normally distributed. Statistical comparison here were performed by Mann–Whitney *U* test and Kruskal–Wallis test. Pearson’s and Spearman’s rank correlation coefficients were used depending on the normality distribution of the data. All statistics were calculated and graphs plotted with GraphPad Prism 6.0 (GraphPad Software, USA); *p* < 0.05 was considered statistically significant.

## Results

### Comparison of maternal characteristics

Comparisons of clinical details between each group, samples used for gene and protein expression (Table 1) and for placental vascular permeability (Table 2), showed no significant difference in maternal age, BMI, gestational age at delivery, placental weight, or baby weight (*p* > 0.05). The HbA_1c_ from all women with GDM showed that glucose levels were under control with both diet and metformin approaches (HbA_1c_ <39.0 mmol/mol or <5.7%).

Birthweight centiles revealed that some babies were above the 95th centile, and therefore classified as large for gestational age (LGA) [33] in both the normal and GDM groups. However, no significant differences were found between groups. There were no correlations between birthweight centiles and occludin protein or gene expression (Pearson’s correlation coefficient *p* > 0.01). In the perfused placentas, no significant correlation was detected (Spearman’s rank correlation coefficient; *p* > 0.01) between vascular leakage and selected variables, which included maternal HbA_1c_, birthweight, birthweight centile and placental weight.

### Lower percentage of junctional occludin-positive vessels in d-GDM

**Fig. 2:**
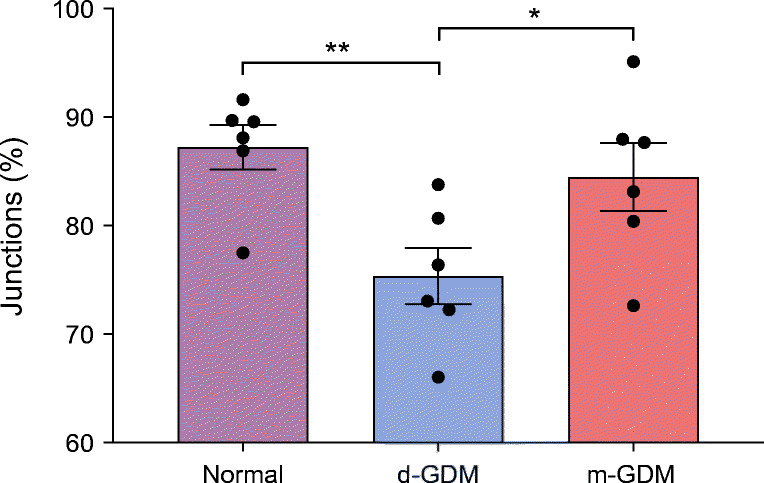
Reduced percentage of occludin-positive vessels in d-GDM pregnancies. Placental samples were taken from normal pregnancies (*n* = 9), and pregnancies complicated with d-GDM (*n* = 7) or m-GDM (*n* = 6) and the endothelial marker PECAM-1 and occludin were immunolabelled prior to systematic counts. The graph shows the percentage of vessels with junctional occludin in the samples from the three groups. Data are presented as mean ± SEM; missing data correspond to outliers. One-way ANOVA between groups (*F*(2, 15) = 5.57, *p* = 0.015); *p < 0.05, **p < 0.01 by post hoc unpaired *t* test

**Fig. 3:**
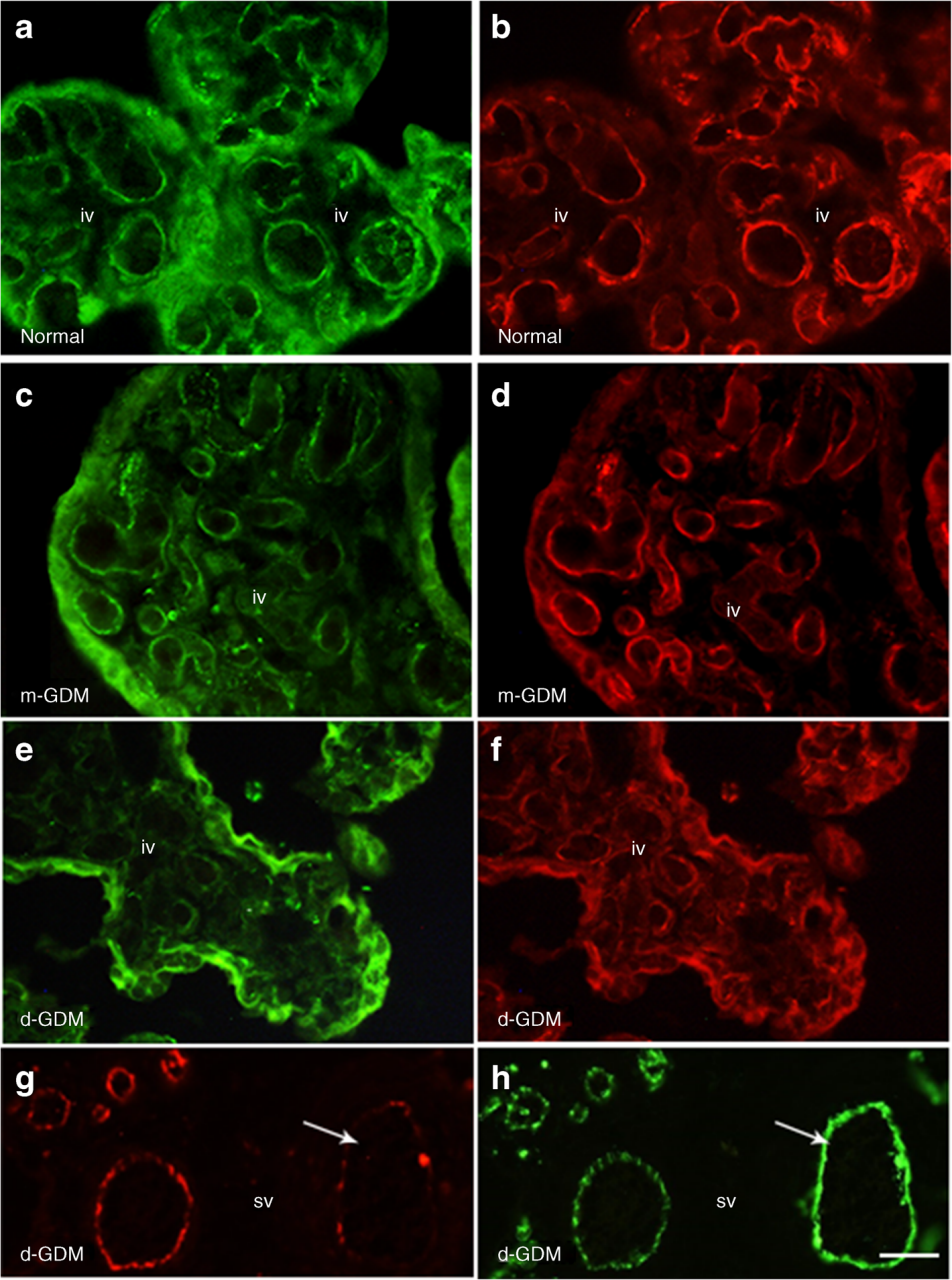
Representative micrographs showing occludin and PECAM-1 localisation in conduit vessels from normal and GDM placentas. Placental samples were taken from normal pregnancies (*n* = 9), and d-GDM pregnancies (*n* = 7) or m-GDM pregnancies (*n* = 6). (**a**, **b**) Full junctional occupancy of occludin (FITC) (**a**) and PECAM-1 (TRITC) (**b**) can be seen in all vascular profiles in intermediate villi (iv) from normal pregnancies. (**c**, **d**) Micrographs showing a similar full occupancy of occludin (**c**) and PECAM-1 (**d**) in intermediate villous vessels from the metformin-treated study group. Figure 3c was taken at a different camera setting (higher gain), to ensure vascular localisation could be seen. (**e**–**h**) Micrographs from diet-controlled GDM placentas showing loss of occludin staining (**e**) from numerous PECAM-1-positive vascular profiles in intermediate villus (**f**). (**g**, **h**) A stem villus (sv) showing occludin immunostaining with TRITC--conjugated anti-rabbit IgG (**g**) and anti-PECAM-1 immunoreactivity with FITC-conjugated anti-mouse IgG (**h**). The arrow points to a large vessel with a reduced number of paracellular clefts showing occludin. Note the presence of occludin in the trophoblast layer in all study groups. Scale bar, 50 μm

The percentage of PECAM-1-positive vascular profiles in stem and intermediate villi showing junctional localisation of occludin were significantly different among the groups (*p =* 0.015) (Fig. 2). In terms of the proportion of occludin-positive vessels, the percentage value was 12% lower in d-GDM samples than in normal samples (*p* = 0.005) and 10% lower in d-GDM samples than m-GDM samples (*p* = 0.049). There was no difference in the percentage of occludin-positive vessels between the normal and m-GDM samples. Figure 3 shows localisation of occludin and PECAM-1 in conduit vessels from normal and GDM placental samples.

### Occludin protein expression is affected in d-GDM

**Fig. 4:**
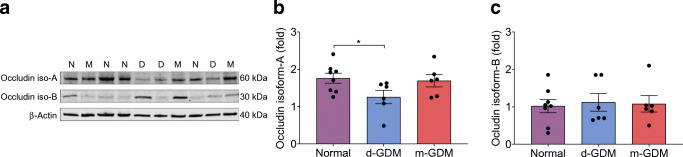
Reduced occludin isoform-A protein expression in human placental tissue from d-GDM pregnancies. Placental samples were taken from normal pregnancies (*n* = 9), and pregnancies complicated with GDM where glucose levels were managed with diet (*n* = 7) or metformin (*n* = 6). (**a**) Representative western blots of occludin isoform-A (~60 kDa) and isoform-B (~ 30 kDa) from placental samples from normal (N), d-GDM (D), and m-GDM (M) pregnancies. β-Actin (~40 kDa) was used as a loading control. (**b**, **c**) Graphs of relative expression of occludin isoform-A (**b**) and occludin isoform-B (**c**) in samples from the three groups. Data are presented as mean ± SEM; missing data correspond to outliers. *p < 0.05 by post hoc unpaired *t* test

Two occludin protein isoforms were detected in human placental samples (Fig. 4a). Occludin isoform-A expression was 29% lower in placental samples from d-GDM pregnancies compared with samples from normal pregnancies (*p =* 0.040) (Fig. 4b). Occludin isoform-B expression was similar among the groups (*p =* 0.938) (Fig. 4c). In placental samples from the m-GDM pregnancies, protein expression of these occludin isoforms was similar to that in samples from normal pregnancies.

### *OCLN* gene expression is affected in d-GDM

**Fig. 5:**
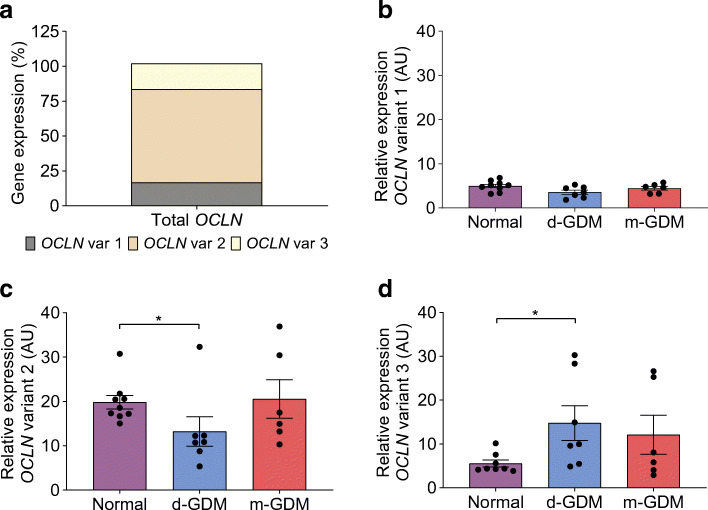
Differential gene expression of *OCLN* in human placental tissue from d-GDM pregnancies. Placental samples were taken from normal pregnancies (*n* = 9), and d-GDM (*n* = 7) or m-GDM (*n* = 6) pregnancies. (**a**) Total *OCLN* gene expression is produced by three *OCLN* splice variants (var) in human placenta from normal pregnancies. (**b**–**d**) Gene expression of *OCLN* variant 1 (**b**), *OCLN* variant 2 (**c**) and *OCLN* variant 3 (**d**) in the three groups. Data are presented as mean ± SEM. *p < 0.05 by Mann–Whitney *U* test

All three predicted *OCLN* splice variants were found in placental samples; *OCLN* variant 2 represents 67% of total *OCLN* gene expression in placental samples from normal pregnancies (Fig. 5a). *OCLN* variant 2 expression was 33% lower in d-GDM samples than in normal placental samples (*p =* 0.016) (Fig. 5c). In the d-GDM samples, *OCLN* variant 3 gene expression was 3.3-fold that in normal placental samples (*p =* 0.020) (Fig. 5d). *OCLN* variant 1 expression was similar in placental samples among the groups (*p =* 0.194) (Fig. 5b). Gene expression of these *OCLN* splice variants in placental samples from the m-GDM pregnancies was similar to that in samples from normal pregnancies.

### Placental vascular permeability is compromised in d-GDM

**Fig. 6:**
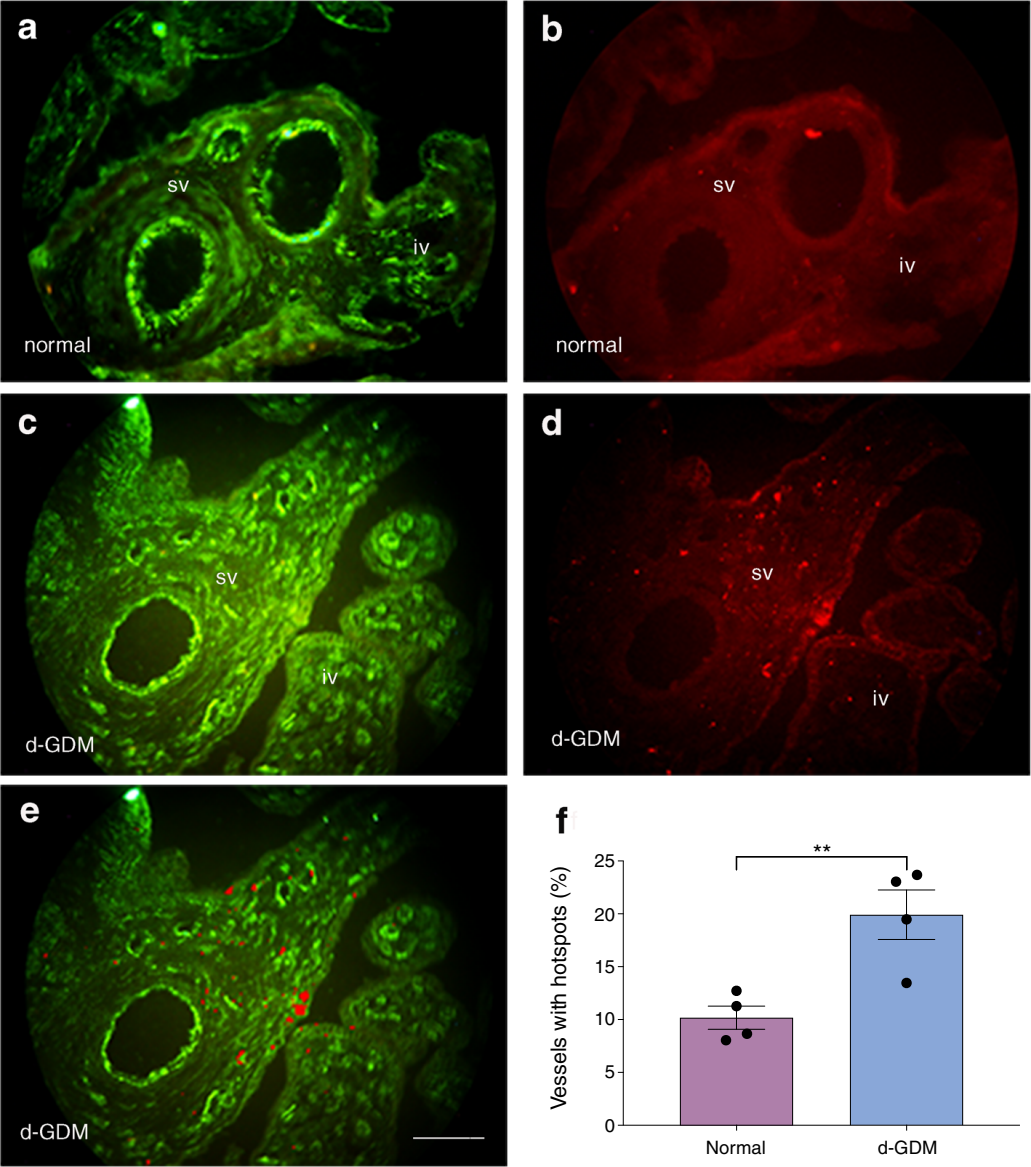
Increased tracer leakage in conduit vessels in placentas from d-GDM pregnancies. (**a**–**e**) Representative micrographs showing PECAM-1 (FITC channel) and dextran tracer (TRITC channel) from perfused placentas from normal pregnancies (*n* = 4), and d-GDM pregnancies (*n* = 4). (**a**, **b**) Fetal vessels in stem villi (sv) and intermediate villi (iv) from normal pregnancies showing no or discreet (<2 hotspots) leakage of 75 *M*_r_ TRITC-dextran. (**c**–**e**) In placentas from the d-GDM group, hotspots of tracer (red) can be seen trapped in the interstitium adjacent to vascular profiles in intermediate and stem villi. (**e**) A composite of the micrographs in (**c**) and (**d**) highlighting hotspots. Scale bar, 50 μm. (**f**) Comparison of percentage of conduit vessels with peri-vascular hotspots between the two groups. Data are presented as mean ± SEM. **p < 0.01 by unpaired *t* test

In placentas from d-GDM pregnancies the percentage of placental conduit vessels (residing in intermediate and stem villi) associated with tracer hotspots was 2.0-fold that in placentas from the normal pregnancy group (*p* = 0.009) (Fig. 6). In the latter, the number of vessels associated with leaks was sparse (Fig. 6a, b), corresponding to 10.18% of the total observed vessels (Fig. 6f). Moreover, the leaks (number of associated hotspots) were discreet. Placental vessels from d-GDM pregnancies showed numerous (>2) hotspots per vessel (Fig. 6c–e) and 19.92% of the total observed vessels showed hotspots (Fig. 6f).

### Increased miR-181a-5p expression in d-GDM placental samples

**Fig. 7:**
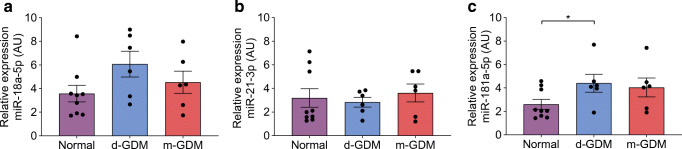
Higher miR-181a-5p expression in d-GDM placental samples. Placental samples were taken from normal pregnancies (*n* = 9), and pregnancies complicated with d-GDM (*n* = 7) or metformin (*n* = 6). The expression of miR-18a-5p (**a**), miR-21-3p (**b**) and miR-181a-5p (**c**) in the three groups. Data are presented as mean ± SEM. *p < 0.05 by post hoc unpaired *t* test

From the five miRNAs screened, three were found in the human placenta: miR-18a-5p, miR-21-3p, and miR-181a-5p (Fig. 7). The expression of miR-18a-5p (*p =* 0.293) (Fig. 7a) and miR-21-3p (*p =* 0.780) (Fig. 7b) was not different among the groups. In placental samples from d-GDM pregnancies, miR-181a-5p expression was 2.0-fold that in placental samples from normal samples (*p =* 0.043) (Fig. 7c). In placental samples from m-GDM pregnancies, expression of these miRNAs was not significantly different from that in samples from normal pregnancies.

### Overexpression of miR-181a-5p downregulates *OCLN* variant 1 and 2 expression in HUVECs

**Fig. 8:**
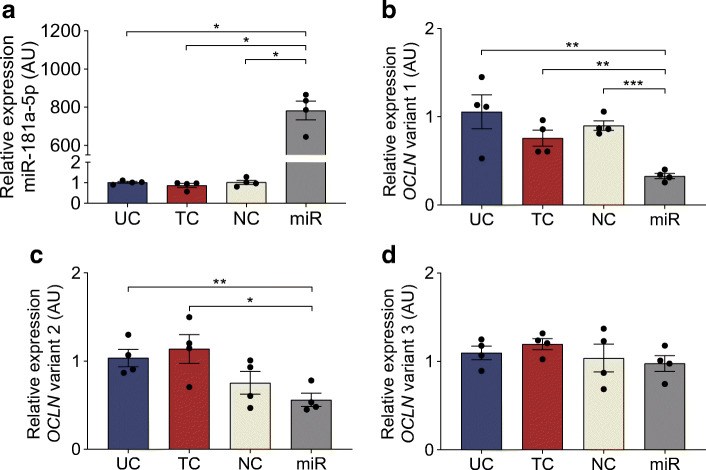
Overexpression of miR-181a-5p downregulates *OCLN* expression. HUVECs isolated from normal pregnancies (*n* = 4) were subject to four transfection conditions for 48 h: untreated control (UC), transfection control (TC), negative control (NC), and miR-181a-5p transfection (miR). (**a**) *miR-181a-5p* expression in HUVECs transfected with the mimic. (**b**–**d**) Expression of *OCLN* variant 1 (**b**), *OCLN* variant 2 (**c**) and *OCLN* variant 3 (**d**). Data are presented as mean ± SEM of four independent experiments, each condition performed in triplicate. *p < 0.05, **p < 0.01, ***p < 0.001 post hoc unpaired *t* test

Overexpression of miR-181a-5p was confirmed by comparing its expression under control conditions with that in miR-181a-5p mimic transfection conditions (*p =* 0.007) (Fig. 8a). The expression of *OCLN* variant 1 was stable among the three control conditions (*p* > 0.05), and 69% lower in miR-181a-5p mimic transfection compared with untreated control (*p =* 0.010), transfection control (*p =* 0.004) and negative control (*p* < 0.001) (Fig. 8b). Likewise, *OCLN* variant 2 expression was stable among the three control conditions (*p* > 0.05), and 46% lower in miR-181a-5p mimic transfection compared with untreated (*p =* 0.009) and transfection control (*p =* 0.018) (Fig. 8c). *OCLN* variant 3 expression was stable between conditions (*p =* 0.509) (Fig. 8d).

### Overexpression of miR-181a-5p decreased endothelial barrier integrity and increased permeability in HUVECs

**Fig. 9:**
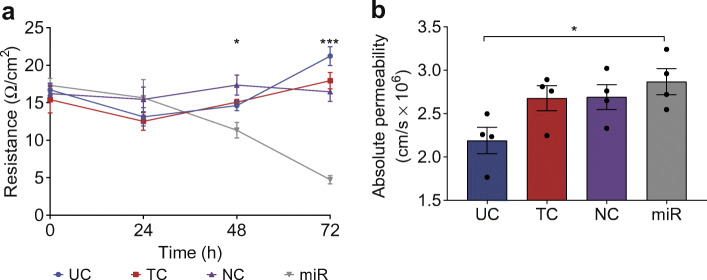
Overexpression of miR-181a-5p reduces endothelial barrier integrity and increases permeability. HUVECs isolated from normal pregnancies (*n* = 4) were subject to four transfection conditions: untreated control (UC), transfection control (TC), negative control (NC) and miR-181a-5p transfection (miR). (**a**) TEER was measured every 24 h after transfection (*t*_0_) for 72 h. (**b**) Tracer leakage was measured 48 h after transfection. Data are presented as mean ± SEM of four independent experiments, each condition performed in triplicate. *p < 0.05, ***p < 0.001 by post hoc unpaired *t* test

TEER was measured to evaluate whether the overexpression of miR-181a-5p would affect endothelial barrier integrity (Fig. 9a). During the different timepoints, resistance was stable among the controls (*p* > 0.05). A reduction of resistance was seen 48 h after transfection, HUVECs overexpressing miR-181a-5p showed 35% lower resistance than controls (*p* < 0.05). This reduction was increased 72 h after transfection, showing 78% lower resistance than controls (*p* < 0.001).

Tracer leakage was measured to evaluate permeability in HUVECs overexpressing miR-181a-5p. At the end of the 2 h experiments, permeability was stable among the controls (*p* > 0.05). In HUVECs overexpressing miR-181a-5p permeability was 1.3-fold that in the untreated control HUVECS (*p* = 0.02) (Fig. 9b).

### VEGF-A protein expression is affected in d-GDM samples

**Fig. 10:**
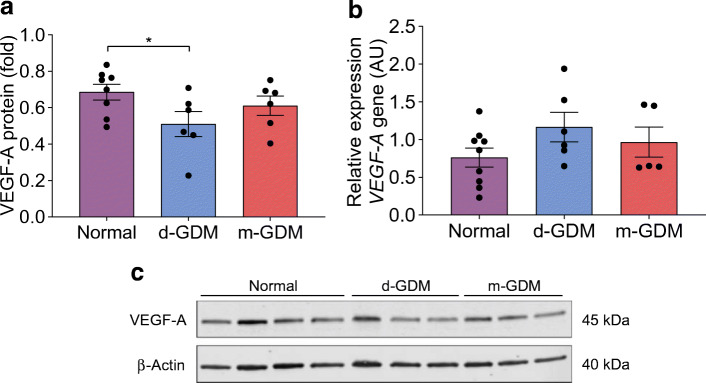
Reduced VEGF-A protein expression in human placental tissue from d-GDM pregnancies. Placental samples were taken from normal pregnancies (*n* = 9), and pregnancies complicated with d-GDM (*n* = 7) or m-GDM (*n* = 6). VEGF-A protein (**a**) and gene expression (**b**) were analysed between groups. (**c**) Representative western blot bands of VEGF-A (~45 kDa) from normal, diet-controlled and metformin-controlled placental samples. β-actin (~40 kDa) used as a loading control. Data are presented as mean ± SEM. *p < 0.05 by post hoc unpaired *t* test

VEGF-A protein expression was 26% lower in placental samples from d-GDM pregnancies than in placental samples from normal pregnancies (*p =* 0.044; Fig. 10a). Placental samples from the m-GDM pregnancies showed stable VEGF-A protein expression compared with samples from normal pregnancies. Total *VEGF-A* gene expression was similar between groups (*p =* 0.370) (Fig. 10b).

## Discussion

This study is the first to show the presence of the three predicted *OCLN* splice variants and their two protein isoforms in human placenta. Our data from the d-GDM group suggests that well-controlled glucose may not be enough to protect against alterations to the *OCLN* gene and occludin protein expression or junctional localisation. Furthermore, we demonstrate that conduit placental microvessels of placental samples from d-GDM pregnancies have a higher percentage of leaky vessels than those in placental samples from normal pregnancies. Interestingly, occludin expression and junctional localisation were similar between placental samples from m-GDM pregnancies and normal pregnancies, and so it is tempting to speculate that metformin may help prevent alterations in occludin expression. Expression of miR-181a-5p was found to be significantly higher in placentas from d-GDM pregnancies. Overexpression of miR-181a-5p in HUVECs from normal pregnancies led to a downregulation of *OCLN* gene expression and reduced endothelial barrier integrity. It is therefore possible that the downregulation of *OCLN* expression in placentas from d-GDM pregnancies may have been epigenetically controlled, in part, by miR-181a-5p expression.

Analyses of junctional localisation of occludin revealed a 12% decrease in occludin-positive vessels in the d-GDM samples. This is in agreement with findings from a previous study showing reduced junctional occludin in placental vasculature in insulin-controlled GDM pregnancies [5]. In the present study, no changes were found in placental samples from the m-GDM group. This marked difference between the two GDM groups reinforces our hypothesis that the type of treatment used to control glucose levels in pregnancies complicated with GDM is an important factor to consider when analysing data. The loss of occludin from tight junction domains may be a consequence of inflammation. Indeed, phosphorylation of junctional adhesion molecules by VEGF-A, resulting in loss of anchorage and internalisation, is a well-established acute response [12, 15, 17, 27]. In our study we did not see increased expression of VEGF-A protein, but, rather, a decrease in the d-GDM placentas. Whether this was due to a reduction in the expression of VEGF-A_165_a or the anti-permeability VEGF-A_165_b splice variant remains to be shown. We have previously shown that perfusion with VEGF-A_165_a results in increased albumin leakage from placental vessels, whilst VEGF-A_165_b reduces paracellular leakage [12]. The altered expression of occludin isoforms seen in placentas from the d-GDM study group suggests that there is an additional mechanism at play.

Our study reports the expression of two occludin protein isoforms (occludin isoform-A and occludin isoform-B) in human placental vessels. As stated above, occludin isoform-A is a fully functional protein that oversees the stability of the membrane [34]; a decrease of this may affect junctional integrity. This hypothesis was confirmed by the 29% decrease seen in d-GDM samples, decreased percentage of occludin-positive vessels and increased vascular leakage in conduit vessels. Protein expression of occludin isoform-A was not altered in the m-GDM group vs the normal group. Curiously, the protein expression of occludin isoform-B, a truncated protein implicated in preventing claudin-4 from attaching to the tight junctions and increasing paracellular permeability [19], was similar among the three groups. Whether this isoform can hamper the barrier integrity function of occludin isoform-A in the placenta requires investigation.

The presence of two occludin protein isoforms suggested that the *OCLN* gene is regulated by alternative splicing. Our previous study confirmed, for the first time, the expression of three *OCLN* splice variants (*OCLN* variant 1, variant 2 and variant 3) in human placental tissue [35]. *OCLN* variant 2 represents ~67% of total *OCLN* expression in placental samples from normal pregnancies. This variant and *OCLN* variant 1 are responsible for the translation of occludin isoform-A. We found that *OCLN* variant 2 expression was decreased by 33% in d-GDM samples. This indicates that the reduction in endothelial junctional occludin from d-GDM samples is not only due to lower protein expression but also the result of a downregulation of *OCLN* variant 2 expression. *OCLN* variant 3 represents ~18% of total *OCLN* expression in normal pregnancy samples, and is translated into occludin isoform-B. Contrary to our results for isoform-B protein expression, in placental samples from the d-GDM group *OCLN* variant 3 expression was 3.3-fold that in samples from normal pregnancies. Therefore, there is still the possibility that the translation of *OCLN* variant 3 may be compromised.

The significant differences in the expression of *OCLN* variant 2 and isoform-A in placentas from the d-GDM group correlate with functional consequences. Perfusion of placental microvascular beds in d-GDM revealed a 100% increase in vessels associated with leaks of a 76 *M*_r_ tracer (slightly larger than albumin) compared with the physiological basal level of leakage exhibited by the vessels in placental samples from the normal pregnancy group (Fig. 6). In the placenta, paracellular permeability to hydrophilic solutes can be induced by VEGF and histamine. We previously reported that perfusion with VEGF-A resulted in reversible TRITC-labelled 76 *M*_r_ dextran leaks and loss of junctional adhesion molecules throughout the placental vascular tree [12], while histamine significantly increased the separation between adjoining endothelial membrane leaflets at tight junction regions (4.1–6.1 nm) but not at adherens junctional zones, leading to increased transport of the smaller molecules cyanocobalamin and EDTA, but not albumin [10]. In the present study, the d-GDM perfused placental leakage pattern suggests the influence of inflammatory conditions and altered structural composition of tight junctions induced by the reduced expression of the transmembrane isoform of occludin. The perfused placentas from the d-GDM group showing altered vascular leaks have not been interrogated with anti-occludin antibodies. The assumption here was that they would have a loss of occludin similar to that observed in the immunofluorescence studies of vascular profiles of the d-GDM study group. Nevertheless, the suboptimal functioning of the d-GDM placental endothelial barrier, i.e. the increased number of conduit vessels showing perivascular tracer hotspots, albeit mild, may have a detrimental effect on total placental function and, concurrently, the developing fetus whose vasculature is in continuum with the placental vasculature. GDM occurs late in gestation, from second trimester onwards, but this shorter exposure to the diabetic milieu still resulted in increased vascular leakage and changes in occludin expression.

Birthweight centiles revealed that some babies were LGA in both the normal and GDM groups. However, no correlations were found between birthweight centiles and occludin protein, gene expression or vascular leakage. Complex interactions of fetal hyperinsulinaemia, hypoxia and overnutrition are involved in the development fetal macrosomia and assessment of measures of these factors was beyond the scope of this study.

Given that *OCLN* expression was affected in the d-GDM group, we explored epigenetic control. Screening for miRNAs that target *OCLN*, revealed that miR-18a-5p, miR-21-3p and miR-181a-5p are expressed in human placenta. The expression of miR-18a-5p and miR-21-3p was similar among groups. However, expression of miR-181a-5p was higher in placental samples from d-GDM pregnancies compared with those from normal pregnancies. Overexpression of miR-181a-5p in HUVECs resulted in an impaired endothelial barrier (78% reduction in resistance), increased 76 *M*_r_ dextran permeability (1.3-fold), as well as the decrease in gene expression of *OCLN* variants 1 and 2. TEER is considered a better in vitro marker of tight junctional integrity in endothelial monolayers with discontinuous ‘kissing’ tight junctions, whilst permeability and transit time of molecules slightly larger than albumin reflect changes in wide zone composition and frequency of tight junctional strand discontinuities along the paracellular cleft [11]. Studies with human glioma endothelial cells showed that overexpression of miR-181a-5p reduces endothelial resistance and the gene expression of tight junction molecules *OCLN*, *ZO-1* and *CLDN-5* [36]. Higher expression of miR-181a was found in non-pregnant individuals with type 1 and type 2 diabetes [37–39] and women affected by a GDM pregnancy tested after 3–11 years [40]. There is evidence that placental miRNAs can traffic to the maternal and fetal circulation [41, 42], thus the possibility remains that the higher expression of miR-181a-5p observed in the d-GDM group could affect maternal and fetal tight junctions. Increased presence of miR-181a-5p in the circulation could lead to downregulation of occludin in different organs and may be behind diabetic retinopathy or nephropathy. The miRNA miR-181a-5p could be one of the factors responsible for the downregulation of occludin in d-GDM placental samples.

Metformin can cross the placenta and has been found in the fetal circulation [43]. It is used for different clinical conditions and reduces insulin resistance. A study using metformin treatment for GDM showed that insulin resistance decreased in blood samples at term compared with baseline [44]. The use of metformin in women with polycystic ovary syndrome (PCO) before conception and during pregnancy reduced serum insulin, insulin resistance and insulin secretion [45]. Several studies have focused on the outcome of offspring from pregnancies treated with metformin. These found no apparent reason for concern on short-term [46–48], or long-term, effects of fetal exposure to metformin [49–51]. In our study, there were no differences in neonatal weight between normal and m-GDM pregnancies; however, the gestational age of the latter was >39 weeks. We did find that m-GDM placentas presented stable expression of occludin gene and protein levels. These results suggest a regulatory effect of metformin over occludin expression in GDM pregnancies but further investigation is required to determine the underlying mechanism.

In conclusion, feto-placental vessels from pregnancies complicated with d-GDM allow the extravasation of normally restricted macromolecules. Beyond phosphorylation of junctional molecules, loss of junctional occludin may be, in part, due to the downregulation of occludin expression and decreased localisation to tight junctions, altered splicing and elevated miR-181a-5p expression. These effects were only visible in the d-GDM group; treatment with metformin appeared to normalise the effects. Our study suggests that lifestyle changes (diet and exercise) in GDM may not be enough to prevent alterations in the expression of occludin and the endothelial barrier integrity. Moreover, these placental changes may be reflecting impaired vascular barrier function in the offspring and their increased vulnerability to inflammatory insults.

## Data Availability

The data are available from the corresponding author on request.

## Abbreviations

C-section: Caesarean section
d-GDM: Diet-controlled gestational diabetes mellitus
GDM: Gestational diabetes mellitus
LGA: Large for gestational age
m-GDM: Metformin-controlled GDM
microRNA: miRNA
PECAM-1: Platelet endothelial cell adhesion molecule 1
PFA: Paraformaldehyde
qPCR: Quantitative real-time PCR
TEER: Trans-endothelial electrical resistance
TRITC: Tetramethylrhodamine isothiocyanate
VEGF: Vascular endothelial growth factor
